# Bioavailability of the Polyphenols: Status and Controversies

**DOI:** 10.3390/ijms11041321

**Published:** 2010-03-31

**Authors:** Massimo D’Archivio, Carmelina Filesi, Rosaria Varì, Beatrice Scazzocchio, Roberta Masella

**Affiliations:** Department of Veterinary Public Health and Food Safety, Italian National Institute of Health, Viale Regina Elena 299, 00161 Rome, Italy; E-Mails: carmelina.filesi@iss.it (C.F.); rosaria.vari@iss.it (R.V.); beatrice.scazzocchio@iss.it (B.S.); roberta.masella@iss.it (R.M.)

**Keywords:** dietary polyphenols, bioavailability, metabolites

## Abstract

The current interest in polyphenols has been driven primarily by epidemiological studies. However, to establish conclusive evidence for the effectiveness of dietary polyphenols in disease prevention, it is useful to better define the bioavailability of the polyphenols, so that their biological activity can be evaluated. The bioavailability appears to differ greatly among the various phenolic compounds, and the most abundant ones in our diet are not necessarily those that have the best bioavailability profile. In the present review, we focus on the factors influencing the bioavailability of the polyphenols. Moreover, a critical overview on the difficulties and the controversies of the studies on the bioavailability is discussed.

## 1. Introduction

Phenolic compounds in foods have attracted great interest since the 1990s due to growing evidence of their beneficial effect on human health. The interest was stimulated mainly by epidemiological studies indicating an inverse association between the intake of foods rich in these compounds and the incidence of diseases, such as cardiovascular disease, diabetes mellitus, and cancer [1–5].

Current dietary advice is that—for optimum health—people should consume at least five portions of fruit and vegetables every day, each portion of at least 80 grams [6,7]. The epidemiological evidence for the benefit of consuming a diet rich in foods containing polyphenols is very strong. On the contrary, the evidence for specific fruit or vegetable, and indeed specific phenolic compounds, is actually less convincing, and the best simple advice that can be given is to recommend as much variety as possible.

Much of the evidence on the beneficial effects of dietary polyphenols is derived from experiments performed *in vitro* or in animal models, and by using concentrations much higher than those generally contained in the human diet. Moreover, often the compounds tested were polyphenol aglycones or their sugar conjugates rather than their active metabolites.

Since the phenolic compounds in dietary sources exhibit potent free radical-scavenging properties, their main role was thought to be as antioxidants involved in protection against lipid peroxidation. However, in the last decade, the mode of action of these compounds has turned out to be more complex than originally expected [8–11]. In fact polyphenols might exert several other specific biological effects. They can inhibit cancer cell proliferation and cholesterol uptake [12,13], modulate different enzymes including telomerase [14] cycloxygenase [15,16] and lipoxygenase [17–19], and interact with several signal transduction pathways [20–24]. Moreover, polyphenols can affect caspase-dependent pathways [25,26], cell cycle regulation [27], and platelet functions [28], and they are also able to prevent endothelial dysfunctions [29].

Even though a compound has strong antioxidative or other biological activities *in vitro*, it would have little biological activity *in vivo* if little or none of the compound gets to the target tissues. The most abundant polyphenols in our diet are not necessarily those that have the best bioavailability profile. Consequently, it is not only important to know how much of a nutrient is present in specific food or dietary supplement, but it is even more important to know how much of it is bioavailable.

The term “bioavailability” was originally used in pharmacology to define the concept of the “rate and extent to which a drug reaches its site of action”. Although several definitions of bioavailability have been suggested, the most appropriate seems to be as that fraction of an ingested nutrient or compound that reaches the systemic circulation and the specific sites where it can exert its biological action [30]. In other words, it simply means how much of the ingested quantity of the polyphenols is able to exert its beneficial effects in target tissues.

To establish conclusive evidence for the effectiveness of polyphenols in disease prevention and human health improvement, it is essential to determine the distribution of these compounds in our diet, estimating their content in each food, and to identify which of the hundreds of existing polyphenols are likely to provide the greatest effects in the context of preventive nutrition. Finally, it is necessary to know the bioavailability of polyphenols and their metabolites, to evaluate their biological activity in target tissues [30].

The main factors influencing the bioavailability of dietary phenolic compounds in humans, and the difficulties and the controversies surrounding the studies aimed at determining the bioavailability of these compounds are the topics of the present review.

## 2. Distribution and Food Content

As far as the distribution is concerned, it is not possible to rank commodities in terms of their production of polyphenols per annum. However the most important food sources are commodities widely consumed in large quantities such as fruit and vegetables, green tea, black tea, red wine, coffee, chocolate, olives, and extra virgin olive oil. Herbs and spices, nuts and algae are also potentially significant for supplying certain polyphenols [31].

Some polyphenols are specific to particular food (flavanones in citrus fruit, isoflavones in soya, phloridzin in apples); whereas others, such as quercetin, are found in all plant products such as fruit, vegetables, cereals, leguminous plants, tea, and wine. However, generally foods contain complex mixtures of polyphenols (see Cheynier 2005 [32] for a review of their structures). Apples, for example, contain flavanol monomers or oligomers, chlorogenic acid and small quantities of other hydroxycinnamic acids, several quercetin glycosides, and 2 glycosides of phloretin and anthocyanins [33]. The profile of polyphenols for all varieties of apples is practically identical, but the concentrations may significantly differ. They may range from 0.1 g total polyphenols/kg fresh weight to 10 g/kg in certain varieties of cider apples [34,35]. Wine also contains a complex mixture of polyphenols, most of which originate in the grape berry. They include flavonols, flavanol, proanthocyanidins, anthocyanins, phenolic acids, hydroxycinnamates and stilbenes [36,37]. Among these compounds, trans-resveratrol is one of the most studied because it is considered to be the main compound responsible for the benefits of red wine on human health [38–42]. However, the concentration of this stilbene in red wine varies considerably. For instance, taking only wine produced with Cabernet Sauvignon grapes in Napa Valley (California, USA) into consideration, the 1989 vintage contained 0.09 mg/L resveratrol whereas the 1994 vintage contained as much as 8.9 mg/l [43,44].

There is much variation also in the composition and concentration of phenolic compounds among virgin olive oils. They contain at least 36 structurally distinct phenolic compounds (see Cicerale S. *et al*. [45] for a detailed review), whose total concentration ranges from 0.02 to 600 mg/kg [46,47]. These discrepancies are explained by multiple factors that have the ability to modify the concentrations of phenolic compounds in foods [37,48].

## 3. Main Factors Affecting the Bioavailability of the Polyphenols

Bioavailability studies are not easy to carry out, since several potentially affecting factors exist, as illustrated in Table 1. These factors may affect bioavailability directly or by decreasing polyphenol content in food.

The methodology generally exploited to study the bioavailability of the polyphenols has to be considered. The *in vivo* approach mostly used is the single-dose design. It involves the intake of one portion of food containing the tested polyphenol. In such a way, the increase in blood concentration is transitional and reflects mainly the ability of the organism to take up the polyphenol from the food matrix. Therefore, the observed increase can have only a minor implication for tissue uptake and bioactivity. On the contrary, under conditions of regular intake, even low amounts of polyphenols can be “repeatedly” absorbed and can significantly increase the concentrations both at plasma and cellular level [49]. Definitive conclusions on the bioavailability and bioactivity of a single phenolic compound are difficult to obtain, because of the synergistic effects of the mixture of polyphenols contained in each food matrix tested. This situation may improve in the future, for example, with the use of isogenic lines of onions that differ only in their quercetin contents [50], allowing comparisons between groups consuming the same food but with different polyphenol contents [5].

### 3.1. External Factors

Numerous factors may affect the content of polyphenols in plants, and the subsequent bioavailability in humans: these factors can be environmental ones, such as sun exposure, rainfall, different type of culture, fruit yield for tree, *etc*. [33,51]. Furthermore, the degree of ripeness affects the concentrations and proportions of the various polyphenols in different ways: generally phenolic acid concentrations decrease during ripening, whereas anthocyanins concentrations increase [52]. It is widely demonstrated that the concentration of phenolic compounds in extra virgin olive oil decreased with the ripeness of olive fruits [51,53]. In particular it has been recently shown that oleuropein, the major olive fruit polyphenol, decreased significantly during the ripeness of olive fruits [56] with a contemporary increase in hydroxytyrosol, one of the principal degradation products of oleuropein [55].

### 3.2. Food Processing Related Factors

***Thermal treatment*** affects the content and, consequently, the amount of the absorbed phenolic compounds in different ways. Thermal processing methods caused significant reduction in total phenolic content and their antioxidant activities in beans [56] and legumes [57]. However, Rocha-Guzman *et al*. [58] reported a significant increase in antioxidant activity in beans (Phaseolus vulgaris L.) cooked at 121 °C. Similarly, Khatun *et al*. [59] observed that total phenolic content and antioxidant activity increased following the heat treatment of several spices.

In extra virgin olive oils, the concentration of hydroxytyrosol, elenolic acid, decarboxymethyl oleuropein aglycon, and oleuropein aglycon decreased more quickly than other phenolic compounds after thermal treatment [60].

In general, ***cooking and the methods of culinary preparations*** have a remarkable effect on polyphenol content [61–67], affecting also their bioavailability and bioactivity.

Miglio *et al*. [62] clearly showed that the physicochemical parameters and the nutritional qualities of vegetables (in particular carrots, courgettes, and broccoli) are modified by common cooking practices. Carrots completely lost their polyphenols after boiling, while steaming and frying had a less negative effect (−43% and −31%, respectively). For broccoli and courgettes, boiling and frying determined a higher loss of total phenolics than steaming. Their results suggest that for each vegetable, a preferential cooking method could be selected to preserve or improve its nutritional qualities.

The common cooking methods applied to artichokes markedly increased the concentrations of caffeoylquinic acids and carotenoids concentrations, particularly upon steaming and boiling, while there was a decrease of the concentration of flavonoids after frying [61]. Phenolic compounds contained in olive oil are subject to degradation upon the application of heat during cooking, although this loss appears to vary among the different phenolic compounds [66,68,69]. In particular, the concentration of hydroxytyrosol in virgin olive oil rapidly decreased after frying. By the end of the first process of frying (10 min at 180 °C), hydroxytyrosol decreased by 40–50% of its original concentration, and after six frying operations less than 10% of the original content of this compound remained [68].

Although consumption of raw vegetables is widely advocated, evidence is emerging that bioavailability of many protective compounds is enhanced when the vegetables are cooked: a significant increase in plasma levels of naringenin and chlorogenic acid was found after the intake of cooked tomatoes compared to the fresh product [70]. The steam-cooking of broccoli results in an increase in the content of polyphenols and in their antioxidant activity [71].

Also ***storage*** affect the content of polyphenols. The storage of apple juices for 11 months resulted in a decrease in phenolic acids from 5% to 21% [72]. A decrease in the content of free p-coumaric acid was also observed in frozen red raspberries [73]. After cold storage, broccoli lost about 75% of its caffeoyl-quinic derivatives and 40–50% of its sinapic acid and feruloyl derivatives [74]. Polyphenol content during storage over seven months in the dark was studied in red wines: the anthocyanins content decreased 88%, while no significant variations occurred in the total flavonol contents [75]. On the other hand, apples (Annurca variety) showed a marked increase in chlorogenic acid after four months storage: from 101 to 144 mg/kg fresh weight [76]; and also the content of phenolic acids increased when carrots were stored under aerobic conditions [77]. Changes occurring in phenolic compounds in virgin olive oil during storage have been reported by several authors. Thirty-four bottles of different quality of extra virgin olive oil (EVOO) were stored for six months in conditions similar to those in consumer sales points: the total polyphenol contents decreased during storage [78]. Under diffused light, about 45% of the phenols were lost in four months [79]. On the other hand, another study showed that the antioxidant activities in EVOOs, during eight months of storage in closed bottles in the dark, were maintained [80]. It has been reported also an increase in hydroxytyrosol and tyrosol content during storage, likely due to the hydrolysis of complex phenols [81].

Technological processes, such as ***homogenization*** of vegetables, could increase the bioavailability of polyphenols by the alteration of the food matrix, as it has been demonstrated for lycopene and for β-carotene, two of the most important carotenoids. In fact it has been shown that tomato puree and paste are more bioavailable sources of lycopene than raw tomato [82].

### 3.3. Food Related Factors

Information on the effect of ***food matrix*** on the bioavailability of phenolic compounds is increasing in recent years. Direct interaction between polyphenols and some food components, such as proteins, carbohydrates, fiber, fat, alcohol, can occur, affecting their absorption [83–87].

Recently, Roura *et al*. [88] evaluated the effect of milk as a food matrix on the bioavailability of epicatechin metabolites from cocoa powder after its ingestion with or without milk in healthy human subjects, concluding that the milk affected the metabolic phenolic profile. However, studies analyzing the effect of milk on the bioavailability of polyphenols from several different foods gave often contradictory results. This fact may be due to a high interindividual variability in the absorption of flavanol in humans, as well as to the small number of subjects selected in the studies [89–93].

Meng *et al*. suggested the possible influence of the matrix sugar content on resveratrol bioavailability, since they showed the lower bioavailability of resveratrol glycosides in grape juice in comparison to the pure aglycones [94]. On the other hand, the absorption of quercetin, catechin, and resveratrol in humans was shown to be equivalent when these polyphenols were administered in three different matrices: white wine, grape juice, and vegetable juice [95].

The possible effect of dietary fat on flavonoid bioavailability was studied by Lesser *et al*. [84], showing that dietary fat content enhances the absorption of flavonoids. Also the fat content of cocoa enhances the digestibility of some phenolic compounds (especially procyanidins) [96]. On the other hand, there may be a physiological interaction between flavonoids and fats, that slows small bowel transit time [97]. This would delay, but not decrease, the absorption of flavonoids, as in the case of anthocyanin-rich strawberries eaten with cream [98]. In contrast, the full-fat yogurt had little effect on the bioavailability of the orange juice flavanones [99].

Phenolic compounds from virgin olive oil have been demonstrated to be highly bioavailable. Hydroxytyrosol and tyrosol are absorbed after ingestion in a dose-dependent way [100,101]. Tuck *et al*. [102] demonstrated the increased bioavailability of hydroxytyrosol and tyrosol when administered as an olive oil solution compared to an aqueous solution.

The possible effect of dietary fiber on quercetin bioavailability was studied by Tamura *et al*. [103]. They reported that pectin might enhance the bioavailability of quercetin from rutin by altering the metabolic activity of the intestinal flora and/or gut physiological function. Recently it has also been shown that the polyphenols associated with dietary fiber are at least partially bioavailable in humans, although dietary fiber appear to delay the absorption [104].

### 3.4. Interaction with Other Compounds

Another factor affecting the bioavailability is represented by ***the interaction with other compounds***. The capacity of the polyphenols and their metabolites to bind proteins must be considered when determining the overall bioactivity. It has been reported the existence of intermolecular bonds between serum albumin and quercetin metabolites, which supports its slow elimination from the body [33]. Similarly, epigallocatechin-3-O-gallate possesses a high affinity for blood proteins [105] which, potentially, could extend its half life in the blood. The binding to albumin and other blood proteins may have consequences for the delivery of the polyphenols and their metabolites to cells and tissues. Also, the interactions with other phenolic compounds with similar mechanisms of absorption can influence the bioavailability of the polyphenols. It has recently been shown that, upon heating, although only a slight decrease in oleocanthal concentration occurred (16%), there was a significant decrease in its biological activity. The authors suggest that this finding could be the result of an antagonist formation, which decreased or masked the biological activity of oleocanthal [106].

### 3.5. Chemical Structure

One of the main factor influencing the bioavailability is the ***chemical structure*** of the compound. In foods most of the polyphenols exists as polymers or in glycosylated forms: the sugar group is known as the glycone and the non-sugar group (the polyphenol) is known as the aglycone. In these native forms the polyphenols cannot be absorbed and must be hydrolyzed by the intestinal enzymes or by the colonic microflora before absorption. Anthocyanins represent an exception, because the intact glycosides can be absorbed and detected in the circulation [107]. The explanation for this may lie in the instability of the aglycone form or in a specific mechanisms of absorption or metabolism for anthocyanins, as suggested by many studies [108,109]. The specific chemical structure of polyphenols as well as the type of the sugar in the glycoside determine their rate and extent of intestinal absorption.

### 3.6. Host Related Factors

The ***host related factors*** affecting the bioavailability can be further subdivided into intestinal factors and systemic factors (Table 1). The intestinal factors represent probably the most important ones. Following the ingestion of dietary polyphenols, the absorption of some but not all components occurs in the small intestine. It is widely accepted that there are two possible mechanisms by which the glycosides could be hydrolysed. The first mechanism involves the action of lactase phloridizin hydrolase (LPH) that is present in the brush-border of the small intestine epithelial cells. LPH has two catalytic sites [110]: one to hydrolyse lactose (LH) and the other involved in the deglycosylation of more hydrophobic substrates. The inhibition of the LH site of the LPH complex markedly reduces deglycosylation of polyphenols [111], showing that the majority of the activity is from the LH domain. The released aglycones may then enter the epithelial cell by passive diffusion as a result of their increased lipophilicity [112].

The second mechanism involves cytosolic β-glucosidase (CBG) that is present within the epithelial cells, where the polar glucosides are transported through the active sodium-dependent glucose transporter SGLT1 [113].

The polyphenols that are not absorbed in the small intestine, reach the colon where they undergo substantial structural modifications. In fact, the colonic microflora hydrolyzes glycosides into aglycones and degrades them to simple phenolic acids [114,115]. This activity is of great importance for the biological action of polyphenols, since active metabolites are produced by the colonic microflora. For instance, daidzein is transformed in its active metabolite (equol) in such a way [116,117]. It is important to underline that a great inter-individual variability in producing these active metabolites exists: for instance, only 30–40% of the occidental people excrete significant quantities of equol after consumption of isoflavones [116,118], while this percentage in Japanese man is about 60% [119]. This variability depends on the genetic characteristic of the subjects: a crucial factor is the presence of specific equol-producing bacteria in the intestine [120]. A critical question is whether a non-equol producer can become an equol producer. If not, a way to circumvent this limitation would be to develop equol as a pharmaceutical or nutraceutical agent. A concerted search for the equol-producing bacteria has more recently led to the discovery of several strains of bacteria that are capable of producing equol *in vitro* when incubated with soy isoflavones [121–123]. This bacterial culture has now been used to produce a natural equol–containing supplement for development as a nutraceutical [124].

**Figure 1.**
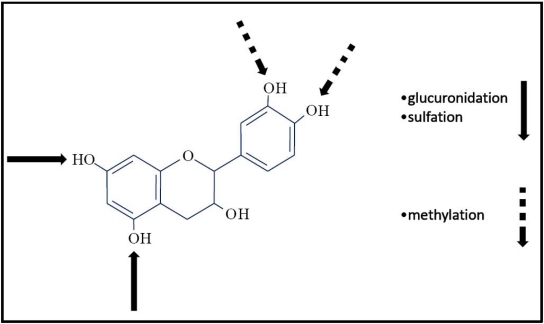
The potential sites of the conjugation process of the polyphenols are schematically illustrated. The *broken arrows* represent the potential methylation sites; the *full arrows* represent the potential glucuronidation and sulfation sites.

Prior to passage into the blood stream, the polyphenols, that now are simple aglycones, undergo to other structural modifications due to the conjugation process [125] that takes place in the small intestine and, mostly, in the liver (Figure 1). The conjugation, that includes methylation, sulfation, and glucuronidation, represents a metabolic detoxication process common to many xenobiotic compounds that restricts their potential toxic effects and facilitates their biliary and urinary elimination by an increased solubility and a higher molecular weight. Glucuronidation is particularly important for increasing molecular weight, necessary for the excretion in the bile [126].

Catechol-O-methyltransferase (COMT) catalyzes the transfer of a methyl group from adenosyl-methionine to polyphenols that contain a diphenolic moiety, such as quercetin, catechin, caffeic acid, and cyanidin. This enzyme is present in a wide range of tissues, but its activity is highest in the liver and the kidneys [127,128]. Sulfotransferases (SULT) catalyze the transfer of a sulfate moiety from phosphoadenosine-phosphosulfate to a hydroxyl group on various substrates, among which polyphenols. Sulfation occurs mainly in the liver [127,129]. Uridine-5’-diphosphate glucuronosyltransferases (UGTs) are membrane-bound enzymes that are located in the endoplasmic reticulum in many tissues and that catalyze the transfer of a glucuronic acid from UDP-glucuronic acid to polyphenols. Glucuronidation of polyphenols first occurs in the enterocytes before further conjugation in the liver [130–132].

Although the process of conjugation on one hand produces active metabolites from some dietary polyphenols, on the other it reduces the total amount of polyphenols in the blood stream, increasing their excretion.

The relative importance of the three types of conjugation vary according to the nature of the substrate and the dose ingested. The balance between sulfation and glucuronidation of polyphenols also seems to be affected by species and sex [64]. It is important to underline that the conjugation mechanisms are highly efficient, and free aglycones are generally either absent, or present in low concentrations in plasma after consumption of nutritional doses. An exception are green tea catechins, whose aglycones can constitute a significant proportion of the total amount in plasma (up to 77% for epigallocatechin gallate) [133].

**Figure 2.**
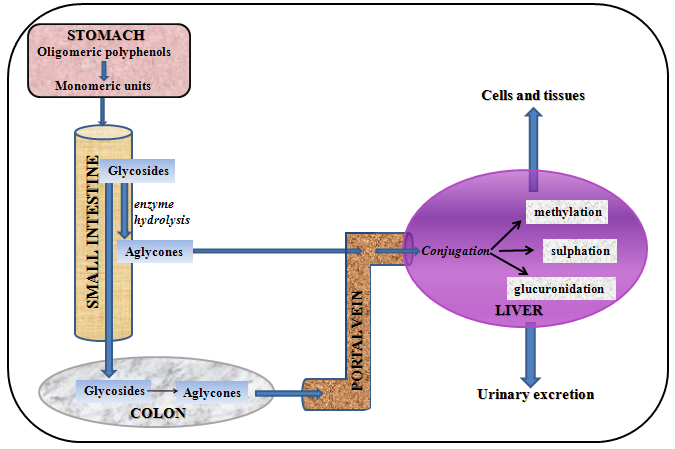
The absorption of dietary polyphenols in humans is schematically illustrated. The polyphenols are extensively modified during the absorption: the glycosides could be hydrolyzed in the small intestine or in the colon, and the released aglycones could be absorbed. Prior to the passage into the blood stream, the polyphenols undergo to other structural modifications due to the conjugation process, mainly in the liver.

Therefore, it is clear that the polyphenols are extensively modified [30], not only in the small intestine and in the colon as it has been discussed above, but also in the liver, where most of the conjugation takes place (Figure 2). Therefore, any single polyphenol generates several metabolites, as many as 20 in the case of quercetin glycosides, although two or three usually dominate [134].

All these modifications deeply affect the biological activity of polyphenols [126,135–139]. Consequently, the compounds that reach cells and tissues are chemically, biologically and, in many instances, functionally different from the original dietary form.

## 4. Limits and Difficulties of the Studies on the Bioavailability of the Polyphenols

The current interest in polyphenols has been driven primarily by many epidemiological studies. Epidemiology is a valuable and necessary tool whose objective is to establish a statistically valid association between the health of a population and one or more factors impacting upon it. However the demonstration of a strong statistical relationship does not establish cause-and-effect, but it may only suggest causality. To demonstrate causality it is essential to define a logical chain of events based on biochemistry, chemistry and physiology, and to support this by intervention studies. Intervention studies are very difficult to carry out, so in many cases the additional support inevitably comes by *in vitro* and animal studies.

A large number of *in vitro* studies, utilizing tissue slices or cultured cells, provided extremely valuable information on the beneficial effects of the polyphenols. However, great care is required when interpreting and extrapolating the data obtained. First of all, often the compounds tested were polyphenol aglycones or their sugar conjugates rather than their active metabolites. Thus many investigators tested the wrong compound when trying to unravel which physiological mechanisms were involved in the health effects of the polyphenols.

Another parameter that should be taken in account in *in vitro* studies is if the test substance is appropriate. For instance, it is clearly unwise to draw hasty conclusions from a study in which a tissue such as liver was directly exposed to tea brew. If the tissue had been the buccal or the gastric epithelium, the experimental conditions might have reflected real life in a better way. So when the test substance is more appropriate to the tissue, valuable data can be generated and the extrapolations that follow are much more reliable.

A very important problem that often compromises *in vitro* studies is the dose applied. The dose used should reflect real life: the tested concentrations commonly range from low μmol/L to mmol/L, while the concentrations of plasma metabolites, after a normal dietary intake, rarely exceed nmol/L [140–142]. Elevated *in vitro* doses can also be used to “force” an outcome but such results must be extrapolated with great care.

The lack of agreement between effective concentrations *in vitro* and those *in vivo* raises an important question. Does the use of these high concentrations in *in vitro* studies make the results irrelevant for the understanding of the biological mechanisms? No clear cut answer is currently available. One may argue that in animals and humans ingesting a specific polyphenol, the cells are consistently exposed to it. So that prolonged exposure can produce significant effects, even though the concentrations are low.

As far as animal studies is concerned, they provide extremely valuable information, and overcome some of the difficulties associated with human intervention trials. For example, animals may be used in a three-generation study, allowing investigation into the reproductive effects and effects on offspring, something requiring over 60 years in humans and obviously totally impractical for many reasons. The data on the tissue distribution of the polyphenols are derived mainly from animal studies [139,143–148], but differences between the human and animal genomes may also lead to potential problems of extrapolation. For example, rodents methylate dietary phenols far more extensively than humans. The three major human metabolites of quercetin glycosides are quercetin-3-*O*-glucuronide, quercetin-3’-*O*-sulfate and isorhamnetin-3-*O*-glucuronyde [134]. On the contrary, quercetin-7-*O*-glucuronide, that represents the major rat metabolite [149], is not present at all in humans [134]. To avoid these problems all together, obviously the best thing to do is to base the studies on *in vivo* experiments.

An important aspect that should be considered in the *in vivo* studies is the methodology generally exploited to study the bioavailability. As we have previously seen, the “*in vivo*” approach mostly used is the “single-dose” design. In such a way, the increase in the blood concentration is transitional and reflects mainly the ability of the organism to take up the polyphenol from the food matrix. Consequently, most of the data from humans presented in the literature on the bioavailability refer only to the release of the polyphenols from the food matrix and their consequent absorption (*i.e.*, concentration in the blood or in the urine). The determination of the bioavailability of the polyphenols in target tissues is much more important than the knowledge of their plasma concentrations: it seems appropriate to consider the plasma response to a phenolic compound as a marker of release from the food matrix and absorption, but only as a ”predictive” indicator of the tissue uptake [30]. Unfortunately, it is still very difficult to evaluate the accumulation and the biological activity of the polyphenols in human tissues, and the *in vivo* studies are still very scarce even though the number of them has rapidly increased over the last few years [150–154].

Another aspect that has to be considered is that the standard metabolites of the polyphenols were rarely available for HPLC analysis. Studies almost invariably involved treatment of samples with glucuronidase/sulfatase enzymes and subsequent quantification of the released aglycones. While, at that time, such studies provided valuable insights, it is important to note that this kind of information on the produced metabolites is very indirect, and quantitative estimates, although precise, are not necessarily accurate, since there are very few data available on the efficiency with which the enzymes hydrolyze the polyphenols and release aglycones [99]. In fact, an *in vivo* study performed in rats, reported that the use of enzyme hydrolysis resulted in an underestimation of isoflavone metabolites [155]. This viewpoint intends to highlight that judgments made on the basis of the published literature of few years ago may have been misleading. The values reported in literature should be reconsidered in the light of the large number of newly identified circulating and excreted metabolites.

## 5. Conclusions

The evaluation of the bioavailability of polyphenols has recently been gaining increasing interest as the food industries are continually involved in developing new products, defined as “functional food”, by virtue of the presence of specific polyphenols. Despite the increasing amount of data available, definitive conclusions on bioavailability of most polyphenols are difficult to obtain and further studies are necessary. At least three critical lines of research should be explored to gain a clear understanding of the health beneficial effects of dietary polyphenols:

1. The potential biological activity of the metabolites of many dietary polyphenols needs to be better investigated. In fact, the identification and the quantification of metabolites currently represents an important and growing field of research.
2. Strategies to improve the bioavailability of the polyphenols need to be developed. Moreover it is necessary to determine whether these methods translate into increased biological activity.
3. Whereas *in vitro* studies shed light on the mechanisms of action of individual dietary polyphenols, these findings need to be supported by *in vivo* experiments. The health benefits of dietary polyphenols must be demonstrated in appropriate animal models of disease and in humans at appropriate doses.

These are important steps for the understanding of the role of the polyphenols in human health, and for optimizing dietary advice to the population.

## Figures and Tables

**Table 1.** Main factors affecting the bioavailability of dietary polyphenols in humans.

**External factors**	Environmental factors (*i.e.*, sun exposure, degree of ripeness); food availability
**Food processing related factors**	Thermal treatments; homogenization; liophylization; cooking and methods of culinary preparation; storage
**Food related factors**	Food matrix; presence of positive or negative effectors of absorption (*i.e.*, fat, fiber)
**Interaction with other compounds**	Bonds with proteins (*i.e.*, albumin) or with polyphenols with similar mechanism of absorption
**Polyphenols related factors**	Chemical structure; concentration in food; amount introduced
**Host related factors**	Intestinal factors (*i.e.*, enzyme activity; intestinal transit time; colonic microflora). Systemic factors (*i.e.*, gender and age; disorders and/or pathologies; genetics; physiological condition)
